# Patient Mistreatment and Depersonalization of Nurses: A Moderated Mediation Model of Person–Organization Fit and Resilience

**DOI:** 10.1155/jonm/7439162

**Published:** 2026-10-29

**Authors:** Wei Yan, Shini Zhang, Di Xiao, Lu Tang, Huan Wang, Xin Du

**Affiliations:** ^1^ School of Economics and Business Administration Chongqing University Chongqing China cqu.edu.cn; ^2^ Medical Insurance Office Hospital of Chongqing University Chongqing China nenu.edu.cn; ^3^ Medical Center of Hematology Military Key Clinical Specialty State Key Laboratory of Trauma and Chemical Poisoning Chongqing Key Clinical Specialty Chongqing Key Laboratory of Hematology and Microenvironment Xinqiao Hospital of Army Medical University (Third Military Medical University) Chongqing China; ^4^ Development and Planning Department Chongqing Medical University Yu Zhong District Chongqing 400000 China cqmu.edu.cn

**Keywords:** depersonalization, nurses, patient mistreatment, person–organization fit, resilience

## Abstract

**Background:**

Mistreatment by patients frequently occurs, threatening the well‐being of nurses and the interests of medical organizations. However, insufficient research has been conducted to determine the mechanisms underlying the association between patient mistreatment and nurses’ depersonalization.

**Objectives:**

This study aimed to investigate the effects of patient mistreatment on depersonalization in nurses, as well as the mediating role of person‐organization fit and the moderating role of trait resilience.

**Methods:**

We conducted a cross‐sectional survey and adopted convenience sampling to recruit nurses from the Hematology Specialist Alliance of Chongqing. The formal online survey was distributed via Wenjuanxing from October to November 2022, and the platform was configured to allow only one submission per IP address. In total, 1627 nurses completed the online survey. The data were analyzed using hierarchical regression analysis and the bootstrap method to test mediation and moderation effects.

**Results:**

Patient mistreatment was negatively associated with person–organization fit (*β* = −0.164, *p* < 0.001) and positively related to depersonalization (*β* = 0.584, *p* < 0.001). Person–organization fit was negatively associated with depersonalization (*β* = −0.368, *p* < 0.001) and partially mediated the relationship between patient mistreatment and depersonalization (indirect effect = 0.0602, 95% CI [0.0346, 0.0882]). The indirect association linking patient mistreatment to depersonalization via person–organization fit differed by resilience level (Index = −0.0208, 95% CI [−0.0382, −0.0076]), with smaller magnitude associations observed at higher resilience.

**Conclusion:**

This study illustrates that nurses who experience patient mistreatment may perceive a decrease in the degree of person–organization fit and become depersonalized; however, nurses with higher resilience are less exposed to such adverse effects.

**Implications for Nursing Management:**

Supportive work environments and resilience training programs should be provided to improve nurses’ coping capabilities when facing patient mistreatment.

## 1. Introduction

A significant majority of healthcare workers experience mistreatment from their patients. The Irish Nurses and Midwives Organization (INMO) reported that at least five nurses per day experienced verbal, physical, and sexual assault in a single month [1]. Patient mistreatment refers to impolite behaviors and negative interpersonal relationships that health workers encounter while providing services [2], including expressions of anger, swearing, insults, rude speech, and physical aggression by patients and their relatives [3]. The adverse consequences of exposure to patient mistreatment have been widely examined, including effects on the emotions and behaviors of medical staff, such as reducing job satisfaction, increasing turnover intention [4], increasing job burnout [3], anxiety, depression, and behavioral stress [5]. Chinese researchers also indicate that patient mistreatment makes nurses feel their dedication goes unrewarded. This sense of inequity creates disappointment toward the nursing profession and diminishes nurses’ occupational commitment [6], and lower nurses’ career aspirations [7]. Furthermore, patient mistreatment positively predicts both job burnout and turnover intention [8]. Among a few indigenous studies on job burnout, research on emotional exhaustion is a prominent research topic. Drawing on conservation of resources theory and goal progress theory, Yan et al. [9] verified that social sharing of negative events mediates the relationship between patient mistreatment and nurses’ emotional exhaustion, with trait resilience and organizational support jointly moderating this pathway. Building upon these findings, Yan et al. [10] further focused on the family‐domain spillover effect. Using perceived organizational support as the moderator, their study revealed that patient mistreatment increases both nurses’ emotional exhaustion and work–family conflict via social sharing of negative work events. Despite growing awareness of these consequences, critical gaps remain in understanding patient mistreatment’s impact on nurses’ depersonalization [11], which is defined as emotional detachment and cynicism toward others and the work environment [12]. Research indicates that nurses’ depersonalization negatively affects their overall nursing quality [13], including low quality of service and endangering patient care and safety [14]. Extant study has identified stress and insomnia [15] as well as perceived stress and job satisfaction [16] as chain mediators between workplace mistreatment and job burnout. However, the mediating mechanisms through which patient mistreatment influences depersonalization (one of the dimensions of job burnout) of nurses have not been fully elucidated. Therefore, this study seeks to fill this gap by combining self‐verification theory and conservation of resources theory and proposing that person–organization (P‐O) fit is a crucial factor in explaining how patient mistreatment affects nurses’ depersonalization.

Furthermore, it is critical to explore under what conditions the negative impacts of patient mistreatment can be effectively mitigated [17]. Previous studies have identified several variables that moderate the relationship between mistreatment and depersonalization, such as social sharing of negative events [18], supervisor support, and job autonomy [19]. However, its boundary conditions still require further in‐depth exploration. Notably, although empirical evidence has shown that resilience serves as a moderator between mistreatment and individual well‐being [20], its moderating effect specifically in healthcare settings—particularly regarding the linkage connecting patient mistreatment to nurses’ P‐O fit and depersonalization—remains underexplored. Hence, the study draws on conservation of resources theory to illustrate that nurses’ resilience may weaken the indirect effect of patient mistreatment on their depersonalization. We respond to the call to explore individual differences (e.g., resilience) as potential moderators between workplace mistreatment and employees’ depersonalization [18].

### 1.1. Theoretical Background and Hypothesis Development

#### 1.1.1. Patient Mistreatment and Depersonalization

Patient mistreatment refers to low‐quality interpersonal treatment that nurses receive from patients and their families [21], which can be viewed as negative feedback on nurses’ service quality, sometimes indicating service failure. Specifically, it includes expressions of anger, swearing, insults, rude speech, and physical aggression [3]. Depersonalization refers to deliberate distancing from the outside world at work, showing indifference and neglect toward others and the work environment [12]. Individuals undergoing depersonalization may experience a desire for disconnection from events in their immediate environment, resembling an external observer rather than actively participating in them [22].

According to self‐verification theory, individuals are inclined to seek information and feedback that aligns with their existing self‐concepts [23]. If feedback aligns with cognitive and affective judgments, it may enhance one’s self‐worth and self‐efficacy. When nurses experience patient mistreatment, external feedback contradicts their self‐concept, leading to a decrease in emotional well‐being and personal resources. In such situations, nurses strive to adjust themselves to restore congruence between their self‐concept and external feedback [23].

Some research regards depersonalization as a self‐defense mechanism that enables nurses to buffer negative emotions such as anxiety and anger caused by patient mistreatment [24]. Therefore, when faced with improper treatment by patients, nurses may experience cognitive dissonance which, in turn, is linked to higher levels of depersonalization, alongside perceptions of elevated adverse outcomes [18]. Thus, we propose the following hypothesis:

 H1: Patient mistreatment is positively associated with nurses’ depersonalization.

#### 1.1.2. The Mediation Role of P‐O Fit

P‐O fit is widely accepted as congruence between the norms and values of organizations and employees [25–29]. Kristof [27] proposed that organizational resources (financial, physical, and psychological) signal the ability of organizations to match individual values. Accordingly, healthcare workers infer whether an organization fits with their personal values based on how the organization treats them as such treatments can, to some extent, demonstrate the values the organization claims to uphold.

Nurses experiencing patient mistreatment tend to perceive that the organization fails to implement effective measures to protect staff from patient mistreatment. This deviates from nurses’ ideal expectation that a qualified workplace should shield employees from abuse. Nurses further recognize that the values advocated by the organization are not reflected in management practices, resulting in perceived value inconsistency and weakened P‐O fit.

To further elaborate on this analysis, we postulate that person–job fit is also likely to influence person–organization fit. Cable and DeRue [30] hypothesized a three‐factor model of subjective fit perceptions and verified that employees distinguish between P‐O fit, needs–supplies (N‐S) fit, and demands‐abilities (D‐A) fit. Although they measured P‐O fit solely as values congruence, they acknowledged that it is also possible that P‐O fit includes a N‐S component. Actually, their results indicated that P‐O fit is positively correlated with N‐S fit (*r* = 0.53) and D‐A fit (*r* = 0.28) in the multiple‐firm sample (Table 2, p880, in Cable & DeRue, 2002).

Patient mistreatment erodes nurses’ perceived complementary fit (person–job fit) by damaging both D‐A fit and N‐S fit. Patient mistreatment elevates emotional and cognitive job demands and creates extra work burdens that exceed nurses’ existing capabilities and limited coping resources, thereby reducing their perceived D‐A fit, which reflects the extent to which employees’ competencies can fulfill job requirements [30]. Meanwhile, frequent patient incivility and abusive behaviors severely frustrate nurses’ core socioemotional needs for workplace safety, interpersonal respect, and professional recognition [31]. When healthcare organizations fail to provide effective protection, supportive resources, or remedial interventions to buffer nurses from patient mistreatment, nurses will perceive poor N‐S fit, as the workplace fails to supply their fundamental psychological and professional needs [27]. According to subjective fit theory, employees infer organizational values and cultural norms based on daily work experiences [32]. The deteriorated D‐A and N‐S fit triggered by negative patient interactions leads nurses to form negative evaluations of their organization’s values and operational philosophies. Consequently, reduced complementary fit further suppresses nurses’ supplementary P‐O fit, manifested as decreased perceived value congruence between individual nurses and their employing medical organizations [30].

Drawing on the self‐verification theory, we propose the following hypothesis:

 H2: Patient mistreatment is negatively associated with P‐O fit.

People are highly motivated to stabilize their self‐concept [23]. To uphold and enhance their self‐concept, employees who strongly align with their organization tend to demonstrate positive emotions and behaviors consistent with high P‐O fit, such as increased job satisfaction and reduced job burnout [33]. However, when P‐O fit is low, nurses may perceive themselves as incompetent and feel that the organization cannot provide the resources that they need. Thus, employees’ psychological needs are not being met, leading to negative emotions and behaviors such as becoming depersonalized and treating others with indifference. Therefore, we propose the following hypothesis:

 H3: P‐O fit is negatively associated with nurses’ depersonalization.

Based on the above analysis, medical workers who endure prolonged patient mistreatment may question their job skills and qualifications. It may also be realized that the organization cannot provide them with the resources needed to fulfill their job responsibilities. Thus, the degree of P‐O fit decreases. To minimize losses and harm, employees will reduce emotional and physical interactions with the work environment, colleagues, and service recipients. Therefore, we propose the following hypothesis:

 H4: P‐O fit mediates the relationship between patient mistreatment and depersonalization.

#### 1.1.3. The Moderating Role of Resilience

Resilience describes one’s ability to manage adverse events and self‐regulate [34, 35]. It allows individuals to swiftly adapt to and recover from unfavorable states by maintaining stable and positive emotions [36]. High resilience buffers negative emotions and facilitates robust psychological recovery, a process mainly driven by positive feedback [37]. Based on the conservation of resources theory, when facing patient mistreatment, employees with high resilience possess greater capabilities and more resources to cope with various challenges and conflicts arising from healthcare service practices. Consequently, they can recover more rapidly from adverse stress and negative emotions and reduce negative feelings toward their work and patients.

In summary, we propose the following hypothesis:

 H5: Resilience moderates the relationship between P‐O fit and depersonalization such that the association is weaker when resilience is higher.
 Combining H1, H2, H3, H4, and H5, we propose that resilience moderates the mediating effect of P‐O fit. Therefore, we propose the following hypothesis:
 H6: Resilience moderates the indirect effect of patient mistreatment on depersonalization through P‐O fit.

The study aimed to investigate the impact of patient mistreatment on nurse depersonalization and explore the mechanism and boundary conditions behind this relationship. Specifically, our study addressed the following questions: Is patient mistreatment related to nurses’ depersonalization through P‐O fit? Can resilience weaken nurses’ depersonalization in response to patient mistreatment? The conceptual model of this study is presented in Figure 1.

**FIGURE 1:**
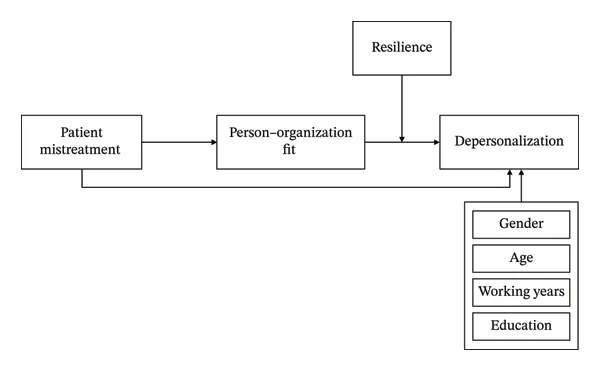
Conceptual model.

## 2. Methods

### 2.1. Design and Setting

This study adopted a cross‐sectional design to investigate the associations between key variables at a single time point. Nevertheless, restricted by cross‐sectional data, the temporal sequence of variables cannot be confirmed. Therefore, this study discusses correlations rather than conducting causal inference. Any claims about causality should be treated with caution.

Data were collected from nurses at major hospitals across Chongqing, China, with recruitment facilitated by the Chongqing Hematology Disease Specialty Alliance Platform. In preparation for the main study, a preliminary survey was conducted to validate the rationality of items and the accuracy of wording. Ninety‐nine clinical nurses from a tertiary Grade A general hospital in Chongqing participated in the pilot test, and 93 valid responses were collected, with a response rate of 93.9%. After the nurses completed the questionnaire, face‐to‐face interviews were conducted with 20 of them to understand their feelings about completing the questionnaire. The formal survey was conducted online via the Wenjuanxing questionnaire website, which was configured to allow only one submission per IP address, from October to November 2022. The questionnaire homepage included statements regarding the study objectives, voluntary participation, and data confidentiality.

### 2.2. Participants

Convenience sampling was used to recruit nurses. The inclusion criteria for recruiting participants were as follows: (1) nurses holding valid practice certificates, (2) working in clinical nursing positions, and (3) providing informed consent and participating voluntarily. The exclusion criteria were as follows: (1) student nurses on rotational practice, (2) student nurses on internship, (3) nursing residents enrolled in training programs, and (4) off‐duty nurses (on leave, sick leave, or attending external training). Regarding the sample sources, this study included 36 hospitals and nine clinical departments. We distributed questionnaires to 2268 nurses who met the inclusion criteria, and 1655 completed questionnaires were received, achieving a response rate of 72.97%. Twenty‐eight incomplete surveys were excluded, resulting in data collected from 1627 nurses.

### 2.3. Measures

The measurements were originally published in English. To ensure the appropriateness of all scales in Chinese, we adopted Brislin’s [38] suggestion and translated the scale forward and backward to ensure Chinese equivalence and prevent semantic bias problems [38]. A bilingual management professor first translated the items from the original English into Chinese. Subsequently, another management professor translated these Chinese‐rendered items back into English. Any discrepancies that emerged during this process were reviewed, discussed, and resolved by both professors.

### 2.4. Demographic Characteristics

Previous research has revealed that gender, age, working years, and education were correlated with depersonalization [39–41], which were used as control variables in this study.

### 2.5. Patient Mistreatment

Patient mistreatment was measured using an 18‐item scale (0 = *never*, 4 = *all the time*) developed by Wang et al. [42]. Minor modifications were made to suit the hospital environment by replacing “customer” with “patient,” as the original scale was designed to assess customer mistreatment. Although originally designed for customer‐related settings, this scale has been extensively applied in other relationally rich work environments, including healthcare. For example, Nielsen and Colbert [43] and Xu et al. [44] successfully adapted this scale to measure patient mistreatment among healthcare workers. Domestic studies have adopted this scale to measure patient mistreatment and obtained favorable reliability and validity outcomes, verifying that the scale retains sound validity and reliability within China’s healthcare context [44, 45]. Sample items included “Patients demanded special treatment” and “Patients believe they are more important than others.” The Cronbach’s alpha coefficient was 0.953.

### 2.6. P‐O Fit

We utilized a 3‐item scale (1 = *strongly disagree* to 5 = *strongly agree*) compiled by Cable and DeRue [30] to evaluate P‐O fit. The scale was empirically tested among Chinese samples, verifying its satisfactory reliability, validity, and local applicability [46]. Items included “My personal values match my organization’s values and culture,” “The things that I value in life are very similar to the things that my organization values,” and “My organization’s values and culture provide a good fit with the things that I value in life.” The Cronbach’s alpha coefficient was 0.959.

### 2.7. Resilience

Resilience was measured using a 6‐item scale (1 = *strongly disagree* to 5 = *strongly agree*) developed by Smith et al. [47]. Chen et al. [48] accomplished the Chinese localization of the scale, and subsequent empirical examinations have validated its favorable reliability and validity [49]. Sample items included “I tend to recover quickly after a difficult situation” and “I have a hard time getting through stressful events.” The Cronbach’s alpha coefficient was 0.826.

### 2.8. Depersonalization

Depersonalization was measured using a 5‐item scale and the intensity scale ranged from 1 (“*very mild, barely noticeable*”) to 7 (“*major, very strong*”) developed by Maslach and Jackson [50]. Song and Zhang [51] translated and developed its Chinese version. Studies have demonstrated that it possesses high reliability and validity and is easy to comprehend, thereby presenting good local applicability [52]. Sample items included “I sometimes feel like I am facing a patient like I am facing an object” and “I’m afraid this job will numb me.” The Cronbach’s alpha coefficient was 0.818.

### 2.9. Data Analysis

Statistical analyses were performed using SPSS Statistics 25.0, PROCESS macro for SPSS, and Amos 26. Normality of continuous variables was assessed using skewness and kurtosis indices. The absolute skewness values for patient mistreatment, P‐O fit, resilience, and depersonalization were 1.431, 0.565, 0.433, and 1.516, respectively. The absolute kurtosis values were 2.693, 0.253, 0.114, and 2.060. All skewness values were < 2, and all kurtosis values were < 7, indicating that the distribution of key variables did not significantly deviate from normality [53]. Descriptive analyses were conducted to describe the demographic characteristics. Categorical variables were presented as frequency (*n*, %), and continuous variables were presented as means and standard deviations.

We conducted a confirmatory factor analysis (CFA) using Amos 26. We used an item‐to‐construct‐balance approach [54] to generate parcels for latent constructs consisting of a large number of items. The parceling method can reduce the ratio of model indicators to observations and random errors manifested in the indicators [54, 55]. CFAs produce more stable and interpretable results when parcels are used to reduce “the number of indicators to an optimal, just identified level” [54].

Hierarchical regression analysis was used to examine the relationships between patient mistreatment, P‐O fit, resilience, and depersonalization. Taking into account the possible deviations from normality assumptions of indirect effect, the mediation and moderated mediation analyses were assessed using the bootstrap method (5000 bootstrapping samples) by PROCESS (Model 4 for mediation and Model 14 for moderated mediation) [56]. The Johnson–Neyman plots in the moderation and moderated mediation analyses were also generated using PROCESS. Gender, age, working years, and education were included as control variables in the subsequent regression analysis, to account for potential confounding effects. Allowing for measurement errors, we also conducted path analysis in structural equation modeling using Amos 26. The results of path analysis were consistent with the findings of hierarchical regression analysis, thus enhancing the robustness and persuasiveness of this study. For brevity, the path analysis is included in the Appendix.

## 3. Results

### 3.1. Characteristics of the Sample

The nurses’ demographic characteristics are presented in Table 1. The mean age of the participants was 31.34 years (SD = 6.018). The mean working years was 9.26 years (SD = 6.398). Most of the participants were female nurses (94.7%). The majority of them were married (62.6%), worked in Level 3A general hospitals (87.5%), and had attended university/college or higher (90.3%).

**TABLE 1:** Nurses’ demographics (*N* = 1627).

Variables	*n* (%)	Mean (SD)
Gender		
Woman	1540 (94.7)	
Man	87 (5.3)	
Marriage		
Unmarried	572 (35.2)	
Married	1019 (62.6)	
Divorced	34 (2.1)	
Bereaved	2 (0.1)	
Education		
Postsecondary	3 (0.2)	
Specialized	154 (9.5)	
Bachelor	1460 (89.7)	
Master	9 (0.5)	
Ph.D.	1 (0.1)	
Age		31.34 (6.018)
≤ 30	824 (50.6)	
30–40	690 (42.5)	
> 40	113 (6.9)	
Tier		
Level 3A general hospital	1424 (87.5)	
Level 3A specialized hospital	31 (1.9)	
Level 3B hospital	22 (1.4)	
Level 2 hospital	150 (9.2)	
Working years		9.26 (6.398)
≤ 5	513 (31.5)	
5–10	575 (35.4)	
> 10	539 (33.1)	

### 3.2. Convergent Validity

We computed the average variances extracted (AVE; > 0.50; [57]) for each construct to verify their convergent validity. As shown in Table 2, the AVE values were 0.671 for patient mistreatment, 0.951 for P‐O fit, 0.539 for resilience, and 0.587 for depersonalization, suggesting good convergent validity of the constructs.

**TABLE 2:** Convergent validity analysis.

Scale	AVE	CR
Patient mistreatment	0.671	0.957
Person–organization fit	0.951	0.983
Resilience	0.539	0.875
Depersonalization	0.587	0.875

Abbreviations: AVE = average variance extracted; CR = composite reliability.

### 3.3. Discriminat Validity

As can be seen from Table 3, the square root of AVE values was greater than intercorrelations among study constructs [57].

**TABLE 3:** Discriminative validity: Pearson correlation and AVE square root values.

Variable	1	2	3	4
1 PM	0.819			
2 P‐O fit	−0.140^∗∗^	0.975		
3 RE	−0.218^∗∗^	0.454^∗∗^	0.734	
4 DE	0.381^∗∗^	−0.332^∗∗^	−0.418^∗∗^	0.766

*Note:* The numbers on the diagonal are the square roots of AVE values.

^∗∗^Correlation is significant at the 0.01 level (two‐tailed).

To evaluate the discriminant validity of our research variables, we conducted a CFA. We used an item‐to‐construct‐balance approach [54] to generate parcels. The indicators of patient mistreatment were divided into three parcels, each consisting of six items. As shown in Table 4, the four‐factor model (*χ* ^2^ */df* = 6.456, RMSEA = 0.058, NFI = 0.965, IFI = 0.970, TLI = 0.962, and CFI = 0.970) showed a better fit than other models, suggesting acceptable discriminant validity. The *χ* ^2^ */df* is higher than the strict cutoff criteria, which may be due to the large sample size (*N* = 1627) increasing the *χ* ^2^ statistic’s sensitivity to minor discrepancies [58, 59]. Even if the model fit is actually reasonable, the chi‐square test may still yield statistically significant results indicating poor fit [60]. Notably, the other fit indices satisfied the recommended criteria, supporting an acceptable fit for the four‐factor model.

**TABLE 4:** Results of confirmatory factor analysis.

Model	*X* ^2^ */df*	RMSEA	NFI	IFI	TLI	CFI
M1	98.949	0.245	0.399	0.402	0.316	0.401
M2	56.228	0.184	0.662	0.666	0.614	0.665
M3	33.327	0.141	0.803	0.808	0.774	0.807
M4	6.456	0.058	0.965	0.970	0.962	0.970

*Note:* One‐factor model M1 (patient mistreatment + person–organization fit + resilience + depersonalization), two‐factor model (patient mistreatment + depersonalization, person–organization fit + resilience), three‐factor model M3 (patient mistreatment, person–organization fit + resilience, depersonalization), and four‐factor model M4 (patient mistreatment, person–organization fit, resilience, and depersonalization).

### 3.4. Common Method Bias

During the measurement process, nurses filled out the self‐reported questionnaire, which may have led to common method bias. To address this problem, we conducted Harman’s single‐factor test. In Table 5, the results revealed that the initial eigenvalues of the six factors exceeded one, and the first factor accounted for only 36.63% of the total variation. Therefore, it is unlikely that this study suffered from significant issues related to common method bias.

**TABLE 5:** Harman’s single‐factor test.

Component	Initial eigenvalues			Extraction sums of squared loadings		
	Total	%	Cumulative %	Total	%	Cumulative %
1	11.721	36.630	36.630	11.721	36.630	36.630
2	4.603	14.383	51.013	4.603	14.383	51.013
3	1.957	6.115	57.128	1.957	6.115	57.128
4	1.741	5.442	62.570	1.741	5.442	62.570
5	1.599	4.997	67.567	1.599	4.997	67.567
6	1.048	3.274	70.840	1.048	3.274	70.840

### 3.5. Correlations of the Constructs

Patient mistreatment was negatively correlated with P‐O fit (*r* = −0.140, *p* < 0.01) and positively correlated with depersonalization (*r* = 0.381, *p* < 0.01). P‐O fit was negatively correlated with depersonalization (*r* = −0.332, *p* < 0.01). Preliminary analyses including descriptive statistics and Pearson correlations are presented in Table 6.

**TABLE 6:** Descriptive statistics and correlations.

Variable	Mean	SD	1	2	3	4
1 PM	1.2634	0.7519	(0.953)			
2 P‐O fit	3.7869	0.8970	−0.140^∗∗^	(0.959)		
3 RE	3.5232	0.6701	−0.218^∗∗^	0.454^∗∗^	(0.826)	
4 DE	2.1460	1.1838	0.381^∗∗^	−0.332^∗∗^	−0.418^∗∗^	(0.818)

*Note:* Coefficients below the diagonal line indicate the correlation coefficients. The numbers on the diagonal line represent Cronbach’s *α* coefficients. RE, resilience; DE, depersonalization.

Abbreviations: PM = patient mistreatment; P‐O fit = person–organization fit.

^∗^ *p* < 0.05.

^∗∗^ *p* < 0.01.

^∗∗∗^ *p* < 0.001.

### 3.6. Hypotheses Testing

We employed demographic information, including gender, age, working years, and education, as control variables.

In Table 7, Model 4 shows that patient mistreatment was positively associated with depersonalization (*β* = 0.584, *p* < 0.001), and the inclusion of patient mistreatment increases *R* ^2^ relative to Model 3 (Δ*R* ^2^ = 0.137), thereby supporting H1. Model 2 reveals that patient mistreatment was negatively associated with P‐O fit (*β* = −0.164, *p* < 0.001), and the inclusion of patient mistreatment increases *R* ^2^ relative to Model 1 (ΔR^2^ = 0.019), supporting H2. In Model 5, P‐O fit was negatively related to depersonalization (*β* = −0.368, *p* < 0.001). Patient mistreatment remained positively associated with depersonalization after controlling for P‐O fit (*β* = 0.523, *p* < 0.001). The inclusion of P‐O fit resulted in further increases in *R* ^2^ relative to Model 4 (Δ*R* ^2^ = 0.076). Bootstrap analysis was utilized with 5000 repeated samples with replacement and a confidence interval (CI) of 95%. In Table 8, the indirect effect was 0.0602 (95% CI [0.0346, 0.0882]), which supports H4.

**TABLE 7:** Results of the mediation analysis.

Variable	Person–organization fit		Depersonalization		
	Model 1	Model 2	Model 3	Model 4	Model 5
*Control variables*					
Gender	0.036	0.024	−0.040	0.003	0.012
Age	0.009	0.005	−0.016	−0.004	−0.002
Working years	−0.002	0.0001	−0.026	−0.034^∗∗^	−0.034^∗∗^
Education	−0.087	−0.088	−0.024	−0.022	−0.054

*Independent variable*					
PM		−0.164^∗∗∗^		0.584^∗∗∗^	0.523^∗∗∗^
Mediator					
P‐O fit					−0.368^∗∗∗^
*R* ^2^	0.003	0.022	0.049	0.186	0.261
Δ*R* ^2^		0.019		0.137	0.076
*F*	1.259	7.229^∗∗∗^	20.924^∗∗∗^	73.887^∗∗∗^	95.598^∗∗∗^

*Note:* RE, resilience; DE, depersonalization.

Abbreviations: PM = patient mistreatment; P‐O fit = person–organization fit.

^∗^ *p* < 0.05.

^∗∗^ *p* < 0.01.

^∗∗∗^ *p* < 0.001.

**TABLE 8:** Bootstrap test for mediation effects.

	Effect	SE	95% CI		Relative effect
			LLCI	ULCI	
Total effect	0.5836	0.0354	0.5142	0.6530	
Indirect effect	0.0602	0.0137	0.0346	0.0882	10.32%
Direct effect	0.5234	0.0340	0.4566	0.5902	89.68%

*Note:* Direct effect: patient mistreatment‐depersonalization; indirect effect: patient mistreatment‐person–organization fit‐depersonalization.

Regression results (Table 9, Model 2) show that P‐O fit was negatively associated with depersonalization (*β* = −0.428, *p* < 0.001), and the inclusion of P‐O fit increases *R* ^2^ relative to Model 1 (Δ*R* ^2^ = 0.105), thus supporting H3. Model 4 demonstrates that the magnitude of the negative association between P‐O fit and depersonalization decreased at higher levels of resilience (*β* = 0.112, *p* < 0.01), and the inclusion of interaction between resilience and P‐O fit increases *R* ^2^ relative to Model 3 (Δ*R* ^2^ = 0.004), which supports H5.

**TABLE 9:** Results of the moderation analysis.

Variable	Depersonalization			
	Model 1	Model 2	Model 3	Model 4
*Control variables*				
Gender	−0.040	−0.025	−0.160	−0.162
Age	−0.016	−0.013	−0.008	−0.007
Working years	−0.026	−0.027^∗^	−0.024^∗^	−0.024^∗^
Education	−0.024	−0.061	−0.074	−0.080

*Mediator*				
P‐O fit		−0.428^∗∗∗^	−0.240^∗∗∗^	−0.228^∗∗∗^
Moderator				
RE			−0.558^∗∗∗^	−0.588^∗∗∗^
PO×RE				0.112^∗∗^
*R* ^2^	0.049	0.154	0.231	0.235
Δ*R* ^2^		0.105	0.077	0.004
*F*	20.924^∗∗∗^	58.886^∗∗∗^	81.100^∗∗∗^	70.855^∗∗∗^

*Note:* RE, resilience; DE, depersonalization.

Abbreviations: PM = patient mistreatment, P‐O fit = person–organization fit.

^∗^ *p* < 0.05.

^∗∗^ *p* < 0.01.

^∗∗∗^ *p* < 0.001.

The simple slopes plot is shown in Figure 2.

**FIGURE 2:**
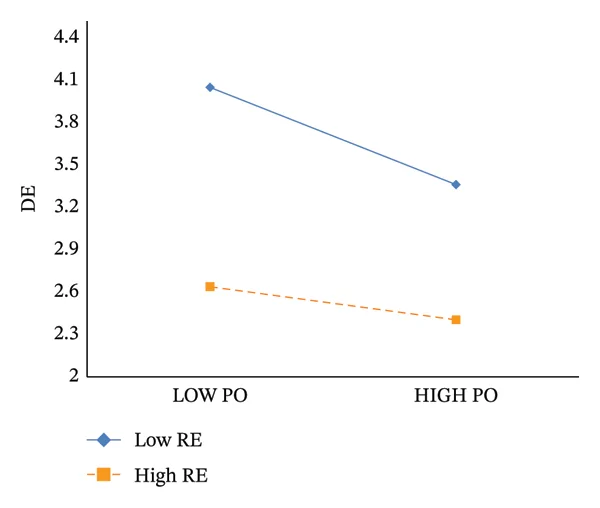
Simple slopes plot depicting the association between person–organization fit and depersonalization at low and high levels of resilience. Note: PO, person–organization fit; DE, depersonalization; RE, resilience.

The Johnson–Neyman diagram includes a simple regression line that specifies how the effect of a predictor on an outcome varies by moderator values, and two 95% confidence bands around that simple regression line. When one of the confidence bands crosses the *x*‐axis, the corresponding value of the moderator is the dividing point between the regions of significance and nonsignificance [61]. In Figure 3, the *x*‐axis displays the standardized resilience value. The critical cutoff value distinguishing significant and insignificant conditional slope is 1.036. Specifically, when standardized resilience is below 1.036 (raw resilience below 4.22), the conditional P‐O fit slope is statistically significant, indicating a significant conditional association between P‐O fit and depersonalization within this range.

**FIGURE 3:**
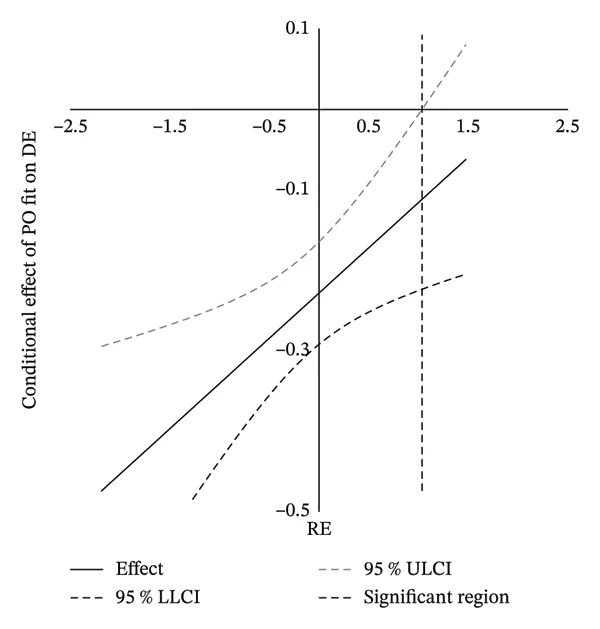
Conditional effect of PO on DE as a function of RE. Note: PO, person–organization fit; DE, depersonalization; RE, resilience.

The bootstrap analysis (Table 10) shows that the index is −0.0208, while the bootstrap 95% CI are [−0.0382, −0.0076]. At low resilience level (M‐1SD), the indirect effect of P‐O fit was significant (effect = 0.0479, 95% CI = [0.0246, 0.0761]). The indirect effect remained significant at resilience level (M) (effect = 0.0340, 95% CI = [0.0173, 0.0541]) and in the high‐resilience group (M + 1SD) (effect = 0.0200, 95% CI = [0.0076, 0.0358]). Therefore, it can be concluded that when employee resilience is higher, the indirect pathways through P‐O fit become smaller. Thus, H6 is supported.

**TABLE 10:** Test of moderated mediation effect.

		Effect/index	Boot SE	(CI)
Indirect effect	M − 1SD	0.0479	0.0132	[0.0246, 0.0761]
	M	0.0340	0.0093	[0.0173, 0.0541]
	M + 1SD	0.0200	0.0072	[0.0076, 0.0358]

Moderated mediation		−0.0208	0.0078	[−0.0382, −0.0076]

Finally, the Johnson–Neyman method was employed to delineate the continuous CIs of the indirect effects. Figure 4 shows the conditional indirect effect of patient mistreatment on depersonalization (via P‐O fit) at different resilience levels. Specifically, it reveals that as resilience increases, the association between patient mistreatment and depersonalization through P‐O fit was weaker.

**FIGURE 4:**
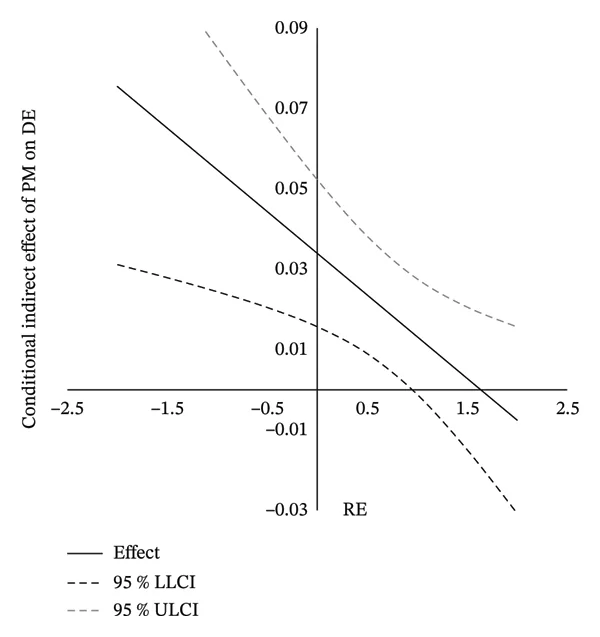
Conditional indirect effect of PM on DE (via P‐O fit) at different RE levels. Note: PM, patient mistreatment; DE, depersonalization; RE, resilience.

## 4. Discussion

### 4.1. Theoretical Implications

First, we delineated how patient mistreatment affects nurses’ depersonalization, thereby enriching research on the outcome variables of patient mistreatment. Previous studies have analyzed the outcome variables of customer mistreatment such as employee turnover intention [62, 63], service disruption [24], job performance, burnout [18, 62], organizational citizenship behavior [64], job satisfaction [63], family well‐being, and psychological detachment [65]. This study contributes to the literature on patient mistreatment by identifying depersonalization as an important consequence of mistreatment.

Second, drawing on self‐verification theory, this study explored the indirect effect of patient mistreatment on depersonalization via P‐O fit, deepening existing knowledge about the mechanism linking customer mistreatment to job burnout. Previous research has primarily viewed customer mistreatment as a stressor through the lens of the conservation of resources theory [3, 62, 65] and the affective events theory [63]. However, this study innovatively applied the self‐verification theory to examine the psychological mechanisms through which patient mistreatment influences nurses’ depersonalization. Specifically, we proposed and verified that patient mistreatment challenges nurses’ self‐concept, leading to negative self‐reflection by nurses [64]. Such self‐reflection diminishes the D‐A fit between the nurse and organization. In the long term, a low level of P‐O fit further decreases self‐verification perceptions [66], thus giving rise to nurses’ depersonalization as a psychological defense mechanism.

Third, we investigated the moderating role of resilience in the association between patient mistreatment and nurses’ depersonalization. Previous studies have suggested that colleague support, self‐efficacy [24], supervisor support, and employee emotion regulation strategies [3] moderate patient mistreatment and work burnout. However, only a few studies have investigated the moderating role of trait resilience. Resilience is often regarded as a resource for coping with stress. Some studies have shown that resilience can mitigate the negative effects of mistreatment on well‐being [20]. However, Yaranon et al. [67] sampled Irish medical staff and found that self‐efficacy, rather than trait resilience, moderates the adverse impacts of insider mistreatment (i.e., coworker and supervisor incivility) on healthcare workers’ affective ill‐being. The cultural background may lead to inconsistent conclusions regarding whether resilience exerts a buffering effect. The present study verifies that resilience can effectively weaken the positive correlation between outsider mistreatment (patient mistreatment) and depersonalization among nurses within the Chinese cultural context. Our findings supplement the academic debate with empirical evidence and expand research on the boundary conditions shaping the emotional reactions of nurses exposed to patient mistreatment.

### 4.2. Implications for Nursing Management

First, this study highlights the role of P‐O fit in the impact of mistreatment on nurses’ depersonalization. Managers are advised to foster a supportive work environment that prioritizes staff well‐being [68]. Establishing a platform for employees to openly express their emotions and promptly address psychological concerns is crucial. This will also signal to employees that the organization cares about individual respect and dignity [69]. Organizations also need to offer recognition and support to healthcare workers, providing skill development and opportunities for external learning to strengthen employees’ self‐affirmation and meet their competency needs. Employees who experience low level of P‐O fit should be provided with job rotation and occupational skills training to enhance their alignment levels.

Second, this study indicated that nurses who experienced high levels of resilience tended to be less vulnerable to patient mistreatment. To this end, managers should introduce employee resilience training programs [70]. Additionally, training programs may be developed to incorporate emotional intelligence education into hospitals’ curricula, which make healthcare workers more resilient and better at managing stress [71]. Furthermore, hospitals may optimize scheduling systems to ensure reasonable work‐rest arrangements for nurses, organize group‐based physical exercise, and leisure stress‐relief activities, so as to continuously improve their psychological well‐being and steadily enhance their resilience and stress tolerance threshold.

### 4.3. Limitations

This study has several limitations. The appropriateness of using cross‐sectional data to test mediation remains debatable. Although we employed procedural and statistical controls and bootstrap methods to strengthen our analysis, the cross‐sectional design precludes establishing temporal precedence among variables, which may limit causal inferences about mediation effects. Future research should consider longitudinal and experimental designs to test the proposed mediation model. This study verifies that P‐O fit partially mediates the relationship between patient mistreatment and depersonalization of nurses. However, the effect size of this mediating pathway is merely 10.32%, indicating relatively weak explanatory power. This finding suggests that other mediating mechanisms not included in the current model (such as emotional labor) might also exist in the process through which patient mistreatment leads to nurses’ depersonalization, which can be further explored in future research. Second, although using self‐report measures may be appropriate because all variables in the current research concern employees’ perceptions, attitudes, and experiences, we acknowledge that same‐source common‐method bias cannot be ruled out. Therefore, we encourage future studies to employ multiple data sources for a more robust examination of the constructs (e.g., manager‐reported or peer‐reported depersonalization) and adopt random staggered layout of items to alleviate the common method bias, rather than relying solely on Harman’s single‐factor test as a post hoc diagnostic. Third, this study was conducted in Chongqing, a southwestern metropolitan area of China, and data were collected amid the COVID‐19 pandemic in 2022, which potentially limits the generalizability of the results. Therefore, future research should be conducted in diverse cultural and temporal settings to boost its external validity. Fourth, the relationship between mistreatment and depersonalization may be more complex than what is analyzed in the current research. Different types of mistreatments, such as verbal versus physical aggression, might affect depersonalization differently. Accordingly, researchers can split patient mistreatment into verbal and physical mistreatment and treat them as independent core explanatory variables, compare the differences in the mediating effect of P‐O fit, and further explore whether trait resilience exerts distinct moderating intensities in the two pathways. Furthermore, future studies could delve into gender differences, focusing on the specific patterns and mechanisms of workplace violence experienced by male nurses [72]. Meanwhile, subsequent research could further identify potential moderators in this relationship. For instance, nurses’ prior work experience, professional training background, and personal coping strategies may moderate the association between patient mistreatment and depersonalization. Finally, given the nested nature of nurses within hospitals, cluster‐robust or multilevel analysis would be preferable. Future studies should consider and weigh the necessity of collecting participants’ organizational affiliation information to account for potential clustering bias.

## 5. Conclusion

This study adopted a self‐verification perspective to investigate the impact of patient mistreatment on nurses’ emotions. The findings demonstrate that nurses who encounter patient mistreatment are more likely to exhibit depersonalization because of their low P‐O fit. Furthermore, this study indicates that resilience, as a moderator, can attenuate the effects of patient mistreatment on depersonalization mediated by P‐O fit. The findings provide healthcare administrators with important practical guidance: They can enhance P‐O fit of nurses by optimizing organizational support environments, such as posting the “Patient Code of Conduct” policy; on the other hand, targeted resilience training programs should be implemented for mitigating mental health risks among medical staff, such as Mindfulness‐Based Stress Reduction (MBSR) programs and Emotional Intelligence development courses. Future studies should adopt multiwave longitudinal designs to replicate the findings and validate the causal relationships in the model.

## Author Contributions

Wei Yan and Shini Zhang designed the study and prepared the first draft of this manuscript. Di Xiao participated in the data analysis. Lu Tang contributed to writing and revising the manuscript. Huan Wang and Xin Du contributed to data collection and analysis.

## Funding

This study was supported by the National Social Science Foundation of China (Grant number: 19BJY052, 22BGL141), National Natural Science Foundation of China (Grant number: 72110107002, 71974021), Natural Science Foundation of Chongqing (Grant number: cstc2021jcyj‐msxmX0689), Fundamental Research Funds for the Central Universities (Grant number: 2022CDJSKJC14), and Chongqing Social Science Planning Project (Grant number: 2018PY76).

## Disclosure

All authors have read and approved the final version of the manuscript.

## Ethics Statement

Ethical approval was obtained from the Ethics Committee of the School of Economics and Business Administration of Chongqing University (IRB No. SEBA201906). The authors explained the research objectives and procedures to all participants, who were assured that their participation in this study was voluntary and anonymous. All procedures performed in this study were in accordance with the ethical standards of the National Research Council and the 2013 Helsinki Declaration. All methods were carried out in accordance with relevant guidelines and regulations. Written informed consent was obtained from all participants.

## Conflicts of Interest

The authors declare no conflicts of interest.

## Figures and Tables

**FIGURE A1:**
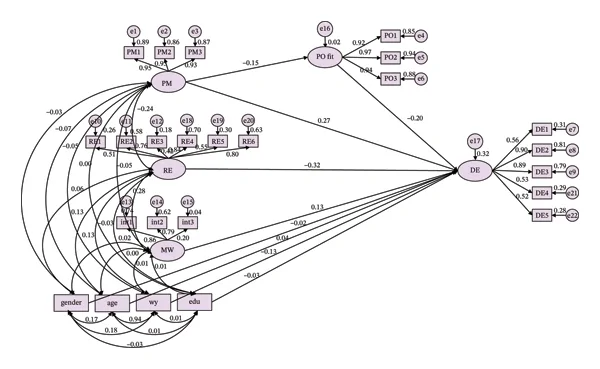
Path analysis to detect the direct and indirect effects of patient mistreatment on depersonalization, mediated by person–organization fit, moderated by resilience. Note: PM, patient mistreatment; PO fit, person–organization fit; RE, resilience; DE, depersonalization; int, PO fit × RE; wy, working years; edu, education.

## Data Availability

The data supporting the findings of this study are available upon request from the corresponding authors. The data are not publicly available due to privacy or ethical restrictions. We affirm that the methods used in the data analyses are suitably applied to our data within our study design and context, and the statistical findings have been implemented and interpreted correctly.

## Appendix A Path Analysis

*Appendix A1: Mediation analysis*: The fit measures for the path model indicate a good fit (*x* ^2^ = 2291.234, *x* ^2^ */df = *10.138, *p* < 0.001, degrees of freedom = 226, NFI = 0.907, CFI = 0.916, and IFI = 0.916, RMSEA = 0.075). The *x* ^2^ */df* is above the strict cutoff criteria due to the large sample size (*N* = 1627). Despite this, the model exhibits acceptable fit when evaluating multiple fit indices. Path analysis was conducted to test the hypotheses. Figure A1 shows the path coefficients for the path analytic model. To construct the latent interaction term between person–organization fit and resilience, we retained all three person–organization fit items and selected the three highest‐loading items from the six resilience items. Following the indicator pairing strategy proposed by Marsh et al. [73], interaction terms were created by multiplying high‐loading indicators with high‐loading indicators, and low‐loading indicators with low‐loading indicators. Table A1 outlines path analysis results testing for the association between patient mistreatment and depersonalization via person–organization fit. Patient mistreatment had a direct effect on person–organization fit (*β* = −0.147, *p* < 0.001) and depersonalization (*β* = 0.274, *p* < 0.001). Person–organization fit had a negative direct effect on depersonalization (*β* = −0.204, *p* < 0.001). The indirect effects of patient mistreatment on depersonalization via person–organization fit was significant (*β* = 0.030, *p* < 0.001).

*Appendix A1: Moderation analysis*: In Table A1, resilience had a direct effect on depersonalization (*β* = −0.318, *p* < 0.001). The path coefficient from the interaction term (resilience × person–organization fit) to depersonalization was significant (*β* = 0.126, *p* < 0.001), indicating that resilience moderated the relationship between person–organization fit and depersonalization.

**Table A1:** The path coefficients for the direct and indirect effects in the path analytic model.

Pathway	*β*	S.E.	*p*	Indirect effect	
				*β*	*p*
PM ⟶ PO	−0.147	0.032	^∗∗∗^		
PO ⟶ DE	−0.204	0.023	^∗∗∗^		
PM ⟶ DE	0.274	0.029	^∗∗∗^	0.030	^∗∗∗^
RE ⟶ DE	−0.318	0.063	^∗∗∗^		
PO^∗^RE ⟶ DE	0.126	0.026	^∗∗∗^		

*Note:* PO, person–organization fit; RE, resilience; DE, depersonalization.

Abbreviation: PM = patient mistreatment.

^∗^ *p* < 0.05.

^∗∗^ *p* < 0.01.

^∗∗∗^ *p* < 0.001.

## References

[1] Irish Nurses and Midwives Organisation(Inmo) , Nurses Make Up Over 60% of Hospital Assaults in June, 2022, https://inmo.ie/Home/Index/217/14004.

[2] Skarlicki D. P. , van Jaarsveld D. D. , and Walker D. D. , Getting Even for Customer Mistreatment: The Role of Moral Identity in the Relationship Between Customer Interpersonal Injustice and Employee Sabotage, Journal of Applied Psychology. (2008) 93, no. 6, 1335–1347, 10.1037/a0012704. 19025251

[3] Goussinsky R. and Livne Y. , Coping With Interpersonal Mistreatment: The Role of Emotion Regulation Strategies and Supervisor Support, Journal of Nursing Management. (2016) 24, no. 8, 1109–1118, 10.1111/jonm.12415. 27406687

[4] Zhao S. , Shi Y. , Sun Z. et al., Impact of Workplace Violence Against Nurses’ Thriving at Work, Job Satisfaction, and Turnover Intention: A Cross-Sectional Study, Journal of Clinical Nursing. (2018) 27, no. 14, 2620–2632, 10.1111/jocn.14311. 29446550

[5] Karaeminogullari A. , Erdogan B. , and Bauer T. N. , Biting the Hand That Heals: Mistreatment by Patients and the Well-Being of Healthcare Workers, Personnel Review. (2018) 47, no. 2, 572–591, 10.1108/pr-03-2016-0054.

[6] Lu J. , Zhou M. J. , and Yang Y. X. , Investigation on the Impact of Patient Mistreatment on Nurses’ Professional Commitment Based on Perceived Social Status and Job Satisfaction, Chinese Journal of Medical Management Sciences. (2022) 12, no. 5, 78–85, 10.3969/j.issn.2095-7432.2022.05.015.

[7] Wang G. Z. , Chen L. , Chi Y. Y. , Zhang R. X. , and Yu S. M. , Research on Status of Career Aspiration of Nurses in Oncology ICU and Its Relationship With Coping Styles After Experiencing Patient Bullyingand Resilience, Occupation and Health. (2023) 39, no. 19, 2597–2602, 10.13329/j.cnki.zyyjk.2023.0487.

[8] Zhou X. , Li X. M. , Yinli Y. T. , Zhang Y. , Li Y. , and Xiang Y. L. , Studying on the Impact of Patients’ Mistreatment on Nurses’ Job Burnout and Intention to Demission, Chinese Health Service Management. (2017) 34, no. 7, 543–545.

[9] Yan W. , Chen X. , Xiao D. et al., Patient Mistreatment, Social Sharing of Negative Events and Emotional Exhaustion Among Chinese Nurses: The Combined Moderating Effect of Organizational Support and Trait Resilience, BMC Nursing. (2024) 23, no. 1, 10.1186/s12912-024-01924-x. PMC11036568 38649852

[10] Yan W. , Cheng Z. , Xiao D. et al., Patient Mistreatment, Emotional Exhaustion and Work-Family Conflict Among Nurses: A Moderated Mediation Model of Social Sharing of Negative Work Events and Perceived Organizational Support, BMC Medical Education. (2024) 24, no. 1, 10.1186/s12909-024-06022-9. PMC11429466 39334234

[11] Zoromba M. A. , Abousoliman A. D. , Zakaria A. M. , El-Monshed A. H. , and El-Gazar H. E. , Mistreatment of Nurses by Patients and Its Impact on Their Caring Behaviors: The Roles of Psychological Detachment and Supervisor Positive Gossip, International Nursing Review. (2025) 72, no. 1, 10.1111/inr.12970. 38683143

[12] Maslach C. and Goldberg J. , Prevention of Burnout: New Perspectives, Applied and Preventive Psychology. (1998) 7, no. 1, 63–74, 10.1016/s0962-1849(98)80022-x.

[13] Salyers M. P. , Fukui S. , Rollins A. L. et al., Burnout and Self-Reported Quality of Care in Community Mental Health, Administration and Policy in Mental Health and Mental Health Services Research. (2015) 42, no. 1, 61–69, 10.1007/s10488-014-0544-6. PMC4171274 24659446

[14] Alquwez N. , Examining the Influence of Workplace Incivility on Nurses’ Patient Safety Competence, Journal of Nursing Scholarship. (2020) 52, no. 3, 292–300, 10.1111/jnu.12553. 32267623

[15] Wang J. , Huang Y. , Wang S. et al., The Impact of Workplace Violence on Job Burnout Among Chinese Correctional Officers: The Chain Mediating Effects of Stress and Insomnia, BMC Public Health. (2024) 24, no. 1, 10.1186/s12889-024-18048-1. PMC10885376 38388879

[16] Zeng C. , Wang F. , Liu W. et al., Relationship Between Workplace Violence and Job Burnout Among Community Nurses in China: A Chained Mediation Modeling Analysis, International Nursing Review. (2025) 72, no. 3, 10.1111/inr.70071. 40678877

[17] Shin Y. and Hur W.-M. , Having Control or Lacking Control? Roles of Job Crafting and Service Scripts in Coping With Customer Incivility, Journal of Occupational Health Psychology. (2022) 27, no. 1, 104–118, 10.1037/ocp0000288. 34323559

[18] Goussinsky R. , The Combined Moderating Effects of Coworker Support and Occupational Coping Self-Efficacy on the Relationship Between Mistreatment by Patients and Burnout, Journal of Aggression, Maltreatment & Trauma. (2020) 29, no. 4, 479–497, 10.1080/10926771.2019.1692982.

[19] Goussinsky R. and Livne Y. , The Role of Coping Resources in the Relationship Between Mistreatment and Job Burnout: Evidence Across Two Healthcare Settings, Journal of Aggression, Maltreatment & Trauma. (2019) 28, no. 10, 1250–1268, 10.1080/10926771.2018.1473905.

[20] Trent S. and Allen J. A. , Resilience Only Gets You so Far: Volunteer Incivility and Burnout, Organization Management Journal. (2019) 16, no. 2, 69–80, 10.1080/15416518.2019.1604199.

[21] Baranik L. E. , Zhu Y. , Wang M. , and Zhuang W. , When Does Witnessing Patient Mistreatment Hurt Nurses’ Performance? Gauging the Moderation Roles of Self-Concern and Other-Orientation, Journal of Managerial Psychology. (2022) 37, no. 2, 170–185, 10.1108/jmp-05-2020-0279.

[22] Kirberg M. and Chadha M. , Depersonalization, Meditation, and The Experience of (No-)Self, Journal of Consciousness Studies. (2024) 31, no. 6, 151–177, 10.53765/20512201.31.5.151.

[23] Swann W. B. , Identity Negotiation: Where Two Roads Meet, Journal of Personality and Social Psychology. (1987) 53, no. 6, 1038–1051, 10.1037/0022-3514.53.6.1038. 3320335

[24] Goussinsky R. , The Moderating Role of Rumination and Social Sharing in the Relationship Between Mistreatment and Service Sabotage and Depersonalization: A Cross-Sectional Study of Hospital Nurses, International Journal of Nursing Studies. (2020) 110, 10.1016/j.ijnurstu.2020.103705. 32731001

[25] Chatman J. A. , Improving Interactional Organizational Research: A Model of Person-Organization Fit, Academy of Management Review. (1989) 14, no. 3, 333–349, 10.5465/amr.1989.4279063.

[26] Bretz R. D. and Judge T. A. , Person-Organization Fit and the Theory of Work Adjustment-Implication, Tenure, and Career Success, Journal of Vocational Behavior. (1994) 44, no. 1, 32–54, 10.1006/jvbe.1994.1003.

[27] Kristof A. L. , Person-Organization Fit: An Integrative Review of Its Conceptualizations, Measurement, and Implications, Personnel Psychology. (1996) 49, no. 1, 1–49, 10.1111/j.1744-6570.1996.tb01790.x.

[28] Verquer M. L. , Beehr T. A. , and Wagner S. H. , A Meta-Analysis of Relations Between Person-Organization Fit and Work Attitudes, Journal of Vocational Behavior. (2003) 63, no. 3, 473–489, 10.1016/S0001-8791(02)00036-2.

[29] Kristof‐Brown A. L. , Zimmerman R. D. , and Johnson E. C. , Consequences of Individuals’ Fit at Work: A Meta‐Analysis of Person-Job, Person-Organization, Person-Group, and Person-Supervisor Fit, Personnel Psychology. (2005) 58, no. 2, 281–342, 10.1111/j.1744-6570.2005.00672.x.

[30] Cable D. M. and DeRue D. S. , The Convergent and Discriminant Validity of Subjective Fit Perceptions, Journal of Applied Psychology. (2002) 87, no. 5, 875–884, 10.1037/0021-9010.87.5.875. 12395812

[31] Hochschild A. R. , The Managed Heart: Commercialization of Human Feeling, 2012, University of California Press.

[32] Sluss D. M. and Ashforth B. E. , Relational Identity and Identification: Defining Ourselves Through Work Relationships, Academy of Management Review. (2007) 32, no. 1, 9–32, 10.5465/AMR.2007.23463672.

[33] Koburtay T. and Alzoubi A. , The Linkages Between Person-Organization Spirituality Fit and Workers’ Psychological Well-Being, The Journal of Social Psychology. (2021) 161, no. 1, 103–118, 10.1080/00224545.2020.1778618. 32538323

[34] Tugade M. M. , Fredrickson B. L. , and Barrett L. F. , Psychological Resilience and Positive Emotional Granularity: Examining the Benefits of Positive Emotions on Coping and Health, Journal of Personality. (2004) 72, no. 6, 1161–1190, 10.1111/j.1467-6494.2004.00294.x. PMC1201429 15509280

[35] Hobfoll S. E. , Conservation of Resources: A New Attempt at Conceptualizing Stress, American Psychologist. (1989) 44, no. 3, 513–524, 10.1037/0003-066X.44.3.513. 2648906

[36] Waugh C. E. , Fredrickson B. L. , and Taylor S. F. , Adapting to Life’s Slings and Arrows: Individual Differences in Resilience When Recovering From an Anticipated Threat, Journal of Research in Personality. (2008) 42, no. 4, 1031–1046, 10.1016/j.jrp.2008.02.005. PMC2711547 19649310

[37] Chang X. , Guo C. , Zhou H. , and Liu L. , Impact of Rumination on Sleep Quality Among Patients With Non-Alcoholic Fatty Liver Disease: A Moderated Mediation Model of Anxiety Symptoms and Resilience, BMC Psychiatry. (2023) 23, no. 1, 10.1186/s12888-023-04572-8. PMC9893673 36732707

[38] Brislin R. W. , Lonner W. L. and Berry J. W. , The Wording and Translation of Research Instruments, Cross-Cultural Research and Methodology Series, 1986, Sage Publications.

[39] Scheibe S. , Spieler I. , and Kuba K. , An Older-Age Advantage? Emotion Regulation and Emotional Experience After a Day of Work, Work, Aging and Retirement. (2016) 2, no. 3, 307–320, 10.1093/workar/waw010.

[40] Sikora J. and Saha L. J. , Vocational Education, Self-Employment and Burnout Among Australian Workers, Australian Journal of Social Issues. (2009) 44, no. 1, 55–72, 10.1002/j.1839-4655.2009.tb00130.x.

[41] Singe S. M. , Cairns A. , and Eason C. M. , Age, Sex, and Years of Experience: Examining Burnout Among Secondary School Athletic Trainers, Journal of Athletic Training. (2022) 57, no. 12, 1094–1099, 10.4085/1062-6050-0731.21. PMC9875710 35192706

[42] Wang M. , Liao H. , Zhan Y. , and Shi J. , Daily Customer Mistreatment and Employee Sabotage Against Customers: Examining Emotion and Resource Perspectives, Academy of Management Journal. (2011) 54, no. 2, 312–334, 10.5465/AMJ.2011.60263093.

[43] Nielsen J. D. and Colbert A. E. , It’s Not Always Sunny in Relationally Rich Jobs: The Influence of Negative Beneficiary Contact, Academy of Management Journal. (2022) 65, no. 6, 1894–1922, 10.5465/amj.2019.1288.

[44] Xu T. , Xu H. , and Wang Z. , Frontline Fallout in Chinese Healthcare: Does Negative Beneficiary Contact Harm Nurses’ Mental Health?, Journal of Advanced Nursing. (2025) 81, no. 10, 6364–6379, 10.1111/jan.16760. 39844521

[45] Yan W. , Bao N. , Zheng S. , Wang H. , Yue D. , and Chen L. , The Impacts of Patient Mistreatment on Healthcare Workers’ Role Behaviors: A Study in Chinese Fangcang Shelter Hospitals, BMC Nursing. (2023) 22, no. 1, 10.1186/s12912-023-01574-5. PMC10668418 38001446

[46] Peng C. , Yuan G. , Xie M. , Zhu L. , and Mao Y. , The Impact of Daily Flow on Employees’ Daily Innovative Behavior: Disentangling the Within-Level Mediation Effect of Job Involvement and the Cross-Level Moderation Effect of Person-Organization Fit, Current Psychology. (2024) 43, no. 29, 24332–24345, 10.1007/s12144-024-06086-2.

[47] Smith B. W. , Dalen J. , Wiggins K. , Tooley E. , Christopher P. , and Bernard J. , The Brief Resilience Scale: Assessing the Ability to Bounce Back, International Journal of Behavioral Medicine. (2008) 15, no. 3, 194–200, 10.1080/10705500802222972. 18696313

[48] Chen W. , Liu J. , Luo J. , and Liu G. Q. , Reliability and Validity of the Chinese Version of Brief Resilience Scale, Chinese Journal of Clinical Psychology. (2020) 28, no. 1, 24–28, 10.16128/j.cnki.1005-3611.2020.01.006.

[49] Wan J. , Pan K. T. , Li Q. , Zeng M. , Zhou X. G. , and Shi K. , Effect of Group Counseling on Improving Resilience of Medical Staff, China Journal of Health Psychology. (2022) 30, no. 4, 557–561, 10.13342/j.cnki.cjhp.2022.04.016.

[50] Maslach C. and Jackson S. , The Measurement of Experienced Burnout, Journal of Organizational Behavior. (1981) 2, no. 2, 99–113, 10.1002/job.4030020205.

[51] Song S. and Zhang L. L. , Reliability and Validity of the Chinese Version of the Maslach Burnout Inventory for Nurses, Guangdong Medical Journal. (2010) 31, no. 4, 501–502, 10.13820/j.cnki.gdyx.2010.04.008.

[52] Li Y. , Liu Y. , Li J. L. et al., The Level and Determinants of Workplace Deviance Behavior Among Nurses, Journal of Nursing Science. (2021) 36, no. 22, 8–11, 10.3870/j.issn.1001-4152.2021.22.008.

[53] West S. G. , Finch J. F. , and Curran P. J. , Structural Equation Modeling: Concepts, Issues, and Applications, 1995, Sage Publications.

[54] Little T. D. , Cunningham W. A. , Shahar G. , and Widaman K. F. , To Parcel or Not to Parcel: Exploring the Question, Weighing the Merits, Structural Equation Modeling: A Multidisciplinary Journal. (2002) 9, no. 2, 151–173, 10.1207/s15328007sem0902_1.

[55] Williams L. J. and O’Boyle E. H. , Measurement Models for Linking Latent Variables and Indicators: A Review of Human Resource Management Research Using Parcels, Human Resource Management Review. (2008) 18, no. 4, 233–242, 10.1016/j.hrmr.2008.07.002.

[56] Zhao X. , John G. , Lynch J. , and Chen Q. , Reconsidering Baron and Kenny: Myths and Truths About Mediation Analysis, Journal of Consumer Research. (2010) 37, no. 2, 197–206, 10.1086/651257.

[57] Fornell C. and Larcker D. F. , Evaluating Structural Equation Models With Unobservable Variables and Measurement Error, Journal of Marketing Research. (1981) 18, no. 1, 39–50, 10.2307/3151312.

[58] Barrett P. , Structural Equation Modelling: Adjudging Model Fit, Personality and Individual Differences. (2006) 42, no. 5, 815–824, 10.1016/j.paid.2006.09.018.

[59] Hair J. F. , Black W. C. , Babin B. J. , Anderson R. E. , and Tatham R. L. , Multivariate Data Analysis, 2010, 7th edition, Prentice Hall, Upper Saddle River, NJ.

[60] Thangamuthu M. and Mohanasundaram T. , Fit Indices in Structural Equation Modeling and Confirmatory Factor Analysis: Reporting Guidelines, Asian Journal of Economics, Business and Accounting. (2024) 24, no. 7, 561–577, 10.9734/AJEBA/2024/V24I71430.

[61] Lin H. , Probing Two-Way Moderation Effects: A Review of Software to Easily Plot Johnson-Neyman Figures, Structural Equation Modeling: A Multidisciplinary Journal. (2020) 27, no. 3, 494–502, 10.1080/10705511.2020.1732826.

[62] Cai D. , Li F. Y. , Feng T. W. , Liu B. , Qi L. , and Men C. H. , Mistreatment from Patients and Nurses’ Career Withdrawal Intention: Does Political Skill Matter?, Asia Pacific Journal of Human Resources. (2022) 60, no. 2, 342–361, 10.1111/1744-7941.12253.

[63] Qi L. , Wei X. , Li Y. H. , Liu B. , and Xu Z. K. , The Influence of Mistreatment by Patients on Job Satisfaction and Turnover Intention Among Chinese Nurses: A Three-Wave Survey, International Journal of Environmental Research and Public Health. (2020) 17, no. 4, 10.3390/ijerph17041256. PMC7068529 32075294

[64] Amarnani R. K. , Restubog S. L. D. , Shao R. D. , Cheng D. C. , and Bordia P. , A Self-Verification Perspective on Customer Mistreatment and Customer-Directed Organizational Citizenship Behaviors, Journal of Organizational Behavior. (2022) 43, no. 5, 912–931, 10.1002/job.2610.

[65] Yang F. , Lu M. Q. , and Huang X. Y. , Customer Mistreatment and Employee Well-Being: A Daily Diary Study of Recovery Mechanisms for Frontline Restaurant Employees in a Hotel, International Journal of Hospitality Management. (2020) 91, 10.1016/j.ijhm.2020.102665.

[66] Kim T.-Y. , Lin X. , and Kim S.-P. , Person-Organization Fit and Friendship From Coworkers: Effects on Feeling Self-Verified and Employee Outcomes, Group & Organization Management. (2019) 44, no. 4, 777–806, 10.1177/1059601117726632.

[67] Yaranon P. , O’Shea D. , Bosak J. , and Sommovigo V. , Insider Versus Outsider Workplace Mistreatment and Their Impact on Affective Ill-Being in Healthcare Professionals: Can Personal Resources Act as Buffers?, Journal of Advanced Nursing. (2024) 80, no. 9, 3705–3720, 10.1111/jan.16120. 38382899

[68] Foster K. , Shakespeare-Finch J. , Shochet I. et al., Psychological Distress, Well-Being, Resilience, Posttraumatic Growth, and Turnover Intention of Mental Health Nurses During COVID-19: A Cross-Sectional Study, International Journal of Mental Health Nursing. (2024) 33, no. 5, 1543–1552, 10.1111/inm.13354. 38747675

[69] Mostafa A. M. S. , Customer Incivility, Work Engagement and Service-Oriented Citizenship Behaviours: Does Servant Leadership Make a Difference?, Human Performance. (2022) 35, no. 1, 31–47, 10.1080/08959285.2021.1998061.

[70] Helmreich I. , Kunzler A. , Chmitorz A. et al., Psychological Interventions for Resilience Enhancement in Adults, The Cochrane Library. (2017) 10.1002/14651858.CD012527.

[71] Shahid R. , Stirling J. , and Adams W. , Promoting Wellness and Stress Management in Residents Through Emotional Intelligence Training, Advances in Medical Education and Practice. (2018) 9, 681–686, 10.2147/amep.S175299. PMC6165721 30310341

[72] Alhalal E. , Workplace Violence and Health Status of Female Nurses: A Cross-Sectional Study, Research in Nursing & Health. (2025) 48, no. 2, 121–132, 10.1002/nur.22434. 39679817

[73] Marsh H. W. , Wen Z. L. , and Hau K. T. , Structural Equation Models of Latent Interactions: Evaluation of Alternative Estimation Strategies and Indicator Construction, Psychological Methods. (2004) 9, no. 3, 275–300, 10.1037/1082-989X.9.3.275. 15355150

